# Medications that interact with direct-acting oral anticoagulant exposure

**DOI:** 10.1007/s12471-021-01617-z

**Published:** 2021-08-18

**Authors:** T. A. C. de Vries, J. R. de Groot

**Affiliations:** 1 Heart Center, Department of Cardiology, Amsterdam University Medical Centers, location Academic Medical Center, Amsterdam, The Netherlands; 2 Department of Cardiology, Rijnstate Hospital, Arnhem, Gelderland, The Netherlands

Direct-acting oral anticoagulant drugs (DOACs) are now the standard of care for stroke prevention in atrial fibrillation [1, 2]. There is mounting evidence indicating that the beneficial effects of DOACs—when compared to vitamin K antagonists—are maintained in patients with cardiovascular comorbidities at the extreme end of the spectrum, including the very elderly, frail patients and those who use multiple drugs concomitantly [3–6].

Many drugs used in daily practice interact with either or both the P‑glycoprotein (P-gp) and/or the cytochrome P450 3A4 enzyme (CYP3A4), which are two of the most important pharmacokinetic substrates for DOACs [2]. Inhibitors of P‑gp and/or CYP3A4 carry the potential to increase DOAC exposure, whereas inducers of these systems can reduce anticoagulant activity [2]. Yet, the concomitant use of such drugs and their interaction with the efficacy and safety of DOACs have not penetrated the literature to the same extent as, for example, cardiovascular comorbidities have.

In this issue of the Netherlands Heart Journal, Harskamp et al. describe the temporal trends of concomitant use of potentially interacting drugs in patients with a new prescription for one of the four available DOACs over five years of follow-up [7].Using a pharmacy database, the authors show that the use of DOACs has quadrupled in Dutch daily practice between 2014 and 2019 (from 8293 to 35,415 patients). Meanwhile, the rate of use of concomitant interacting medications remained more or less stable within that time frame [7].

These findings are of interest, because there is convincing evidence that indicates a weak and inverse relationship between DOAC exposure and thrombosis, and a strong and positive association with exposure and bleeding events [8–12]. As a consequence, the use of drugs that have the potential to significantly increase or decrease DOAC exposure may affect clinical outcomes [2]. This underscores the need for physicians to caution the use of potent interacting drugs, and to find alternatives whenever possible. Below, we give some considerations with regard to the findings of Harskamp et al., and their implications for routine practice.

First, in the study by Harskamp et al., all interacting drugs were considered uniformly, and the numbers and proportions of their users were determined for each DOAC [7]. However, the extent to which DOAC exposure may be affected by the interacting drugs analysed is often unknown, and when known, often varies widely among the individual DOACs [2]. This is because the pharmacokinetics of each DOAC, including their dependency of CYP3A4 for elimination, are not homogenous [2]. In line with this notion, some of the drugs discussed at length in the manuscript, such as digoxin and atorvastatin, reportedly have no notable effect on the exposure of any of the four DOACs [2, 7].

Second, their manuscript does not provide information on the direction of the interaction (increased versus decreased exposure) per concomitant drug [7]. The potential risk for either underexposure or overexposure, and thus clinical outcomes, can only be assessed when this direction is taken into consideration. Although, to our knowledge, there is sparse data on the differential effect of combinations of interacting drugs, it stands to reason that the risk for DOAC levels that are too low or too high is likely to fluctuate considerably with different combinations.

In conclusion, the contribution of Harskamp et al. [7] provides an overview of the magnitude of use of potentially interacting drugs. Their finding that close to 80% of patients used one, 20% of patients two, and approximately 4% of patients three or more potentially interacting drugs, is both concerning and very relevant. Importantly, many of these drugs are often not, or not only, prescribed by a cardiologist (Dutch general practitioners are allowed to prescribe DOACs as well), which further underlines the need of careful assessment of ongoing prescriptions in patients using DOACs.

Future studies should also include subgroup analyses on patients who use DOACs who, on clinical grounds other than simultaneous use of drugs that are known P‑gp and/or CYP3A4 substrates, are deemed to be at a high risk of thromboembolic or bleeding events [2]. Particularly these patients are likely to benefit from cautioning use of interacting medications.
